# *Allolobophora caliginosa* as bioindicator for chitosan–saponin–bentonite nanocomposite contaminated soil

**DOI:** 10.1038/s41598-025-07708-w

**Published:** 2025-07-02

**Authors:** Ghada Mohamed Ahmed, Nesma A. Mostafa, Manal El-Garhy, Sohair R. Fahmy, Ayman Saber Mohamed, Sara Bayoumi Ali

**Affiliations:** Zoology Department, Faculty of Science, Cairo University, Giza, 12613 Egypt

**Keywords:** *Allolobophora caliginosa*, Bioindicator, Chitosan, Saponin, Bentonite, Nanocomposites, Ecology, Zoology

## Abstract

The usage of nanocomposites in water treatment has risen, resulting in their leaking into the soil, which is a major environmental concern. Earthworm (*Allolobophora caliginosa*) is used as a bioindicator that can accumulate most pollutants, even if they are present in low concentrations. The present study aimed to use earthworms as biological indicator for chitosan–saponin–bentonite nanocomposite (CSB NCs) in the soil. Earthworms were exposed to CSB NCs (0, 0.025, 0.05, 0.1, and 0.15 mg/500 g soil) for 7 consecutive days. CSB NCs induced significant damage and instability of the lysosomal membranes in coelomocytes in a dose-dependent manner. The exposure to CSB NCs resulted in a notable change in earthworm biochemical levels. Light microscopy revealed histological damage in the body wall and intestine of earthworm exposed to CSB NC. In addition, scanning electron microscopy showed morphological alterations in the anterior, dorsal, and ventral parts of the earthworm as well as in the anal region because of exposure to CSB NC. The present study demonstrated that earthworms exposed to CSB NCs had a depletion in antioxidants and presented histological alterations especially in high doses of nanocomposite. Also, treated earthworms showed substantial alterations in the surface topography. Exposure to CSB NC caused physiological and histological alteration in earthworms. This study emphasizes the urgent need to evaluate the environmental safety of nanocomposites used in water treatment.

## Introduction

Pristine soils and unpolluted water sustain all life on Earth and are vital for human health^1^. The persistent escalation of environmental pollution has emerged as a paramount issue for both the scientific community and the general populace during the past few decades^2,3^. Water and soil contaminants constitute two principal kinds of environmental pollution^4^. Chemical contamination of soil, water, air, and food is a significant environmental hazard, resulting in approximately 9 million premature fatalities globally^5,6^. Exposure to pollutants and contaminants can arise via water or soil, which may be tainted either naturally or by anthropogenic activity^7^.

Nanotechnology offers promising solutions via the tailored creation of nanoparticles and nanocomposites for water purification applications^8,9^. Nanoparticles, less than 100 nm, have physicochemical features such as large surface area and strong penetrating ability that make them useful in industry and medicine^10–12^. Nanocomposite biomaterials are a novel category of materials that integrate a biopolymeric and biodegradable matrix with bioactive, easily resorbable nano-sized fillers^13^. A composite is a combination of two or more different nanomaterials to achieve superior properties. The contamination of water sources with heavy metals is an important problem that can affect people’s health. As technology advances, an increasing number of both commercial and non-commercial approaches are being developed to address this problem^14^. So, using nanocomposites has been shown to be a highly effective method for treating wastewater^15^.

There are several nanocomposites, such as chitosan–saponin–bentonite (CSB), which has the highest removal efficiency toward molybdenum and chromium for water treatment^16^. Natural bentonite exhibits high hydrophilicity and readily interacts with water molecules, hence impeding the diffusion and adsorption of organic compounds on its surface^17^. Nonetheless, the utilization of bentonite presents disadvantages, including diminished adsorption efficiency, absence of selectivity, and poor binding coefficients^18^. Chitosan and its composites have emerged as attractive adsorbents due to their affordability, abundance, presence of amino and hydroxyl groups, and capability to eliminate numerous pollutants from wastewater^19,20^. Nonetheless, other obstacles related to its practical application encompass insufficient selectivity, diminished mechanical strength, and solubility in acidic environments^21^. Saponin functions as a surface-modifying agent, and its incorporation results in surface modification via the chemical interaction of its two acyl groups^22^.

During the water treatment process, there is a leakage of chitosan, saponin, and bentonite nanocomposite into the soil, causing harmful impacts on the soil creatures, as well as their probable influence on human health^23^. Upon entering the soil system, nanoparticles may undergo various bio/geo-transformations that ultimately influence their bioavailability and toxicity^24^. Such reactions can lead to toxicity, induce oxidative stress, and be assimilated by plants, thereby posing a potential risk to human health through transfer within the food chain^25,26^. Nevertheless, numerous research studies have been undertaken on nanomaterials; however, their behavior within soil pores, interactions with soil biota, adsorption onto mineral particles, and interactions with pollutants remain unexplored^27^.

Bioindicators such as plants, plankton, animals, and bacteria are used to monitor the health of the natural ecosystem in their habitat. They are used to measure environmental health and biogeographic changes that occur in the environment^28^. The earthworm, *Allolobophora caliginosa*, plays an important role as a bioindicator for soil physical characteristics and processes such as mineral turnover, water permeability, and aeration^29^. They also play an important role in the soil’s nutrient recycling and distribution activities^30^. Therefore, the current study aims to evaluate the toxicological effect of CSB nanocomposite on the earthworm (*Allolobophora caliginosa*) as a bioindicator for soil quality.

## Material and methods

### Preparation and characterization of CSB NC

Chitosan with medium molecular weight and saponin were purchased from Sigma-Aldrich. The nanocomposite, CSB, was prepared by the suspension method. 1 g of saponin and 10 g of bentonite were mixed in 100 ml of distilled water. The mixture was stirred for 30 min at 80 °C. After that, it had cooled to room temperature and centrifuged for 15 min at a speed of 3000 rpm. The obtained bottom solid was dried at 105 °C in an oven. The saponin–bentonite was dried and sieved to a particle size of 200 mesh. 1 g of both saponin–bentonite and chitosan were added to 1000 ml of 1 M acetic acid (CH_3_COOH), then dried in a 60 °C oven overnight and stored in a desiccator until used^14^.

The crystallographic characteristics of CSB NC were investigated using a PANalyticalX’Pert X-ray diffractometer (XRD) fitted with a nickel filter and copper (Cu) Ka radiation as an X-ray source. The particle size was determined using Scherrer’s formula, which is as follows: d = Kλ/β cos θ.

Where d is the crystalline size, K = 0.89 is the form factor, k is the X-ray wavelength of Cu Ka radiation (0.154 nm), θ is the Bragg diffraction angle, wavelength λ (nm), and β is the whole width at half maximum of the corresponding diffraction peak.

The shape and size of synthesized CSB NC were determined using transmission electron microscopy (TEM) at an accelerated voltage of 120 kV (JEM-JEM 2100F; JEOL Ltd, Tokyo, Japan, Electron Microscopy Unit, Faculty of Agriculture, Cairo University).

The functional groups of CSB NC were examined using Fourier transform infrared spectroscopy (FT-IR) to determine any chemical interactions between CSB NC and other components. The CSB NC was freeze-dried, mixed with a potassium bromide (KBr) pellet, and pressed into a disc form. Then, an FT-IR spectrophotometer (JASCO FTIR-6200, JASCO International Co., Ltd.-Japan) was used to record IR spectra (range 4000–400 cm^−1^).

### Experimental design

Soil that was pesticide-free and devoid of any contamination was used. The soil was divided into 5 groups in plastic boxes, each containing 500 g of soil and being irrigated with 10 ml of dechlorinated tap water every day starting from the second day of the experiment. The moisture content of each group was 74.05%, 68.97%, 68.61%, 71.67%, and 70.1%, respectively. The pH content of the soil was 8–8.5, and the temperature was 20–21 °C^31^.

One hundred twenty-five adult earthworms were obtained from the faculty of agriculture, Cairo University; the average weight of each worm is 1 g. International standards for the treatment and use of laboratory animals were followed during the conduct of every experiment. Earthworms were assigned to the 5 groups of soil (n = 25 earthworms/group) (one control and four treated groups). 2.5 g of dry grass was added three times a day for the whole duration of the experiment (7 days). The soil was then mixed with CSBNC to get the concentrations of 0, 0.025, 0.05, 0.1, and 0.15 g per 500 g of soil (one control and four treated groups). After 7 days, the worms were collected and used for neutral red analysis, biochemical and oxidative stress markers, and light and scanning electron microscope examination.

### Evaluation of neutral red retention time assay

For neutral red retention time, earthworms were washed in dechlorinated tap water for 30 min. They were placed individually in Petri dishes containing 1 ml physiological saline at 45 °C for 2 min to expel coelomic fluid with coelomocytes. Then 10 µl of extrusion fluid and 10 µl of neutral red were incubated in vitro in a hemocytometer for 1.5 h. The total number of extruded coelomocytes (stained and unstained) was counted at 5-min intervals, and cells were counted until 50% of the cells were stained^32^.

### Biochemical and oxidative stress assessment in earthworms

Control and treated worms were homogenized in phosphate buffer (50 mM, pH 7.4) and centrifuged at 4000 rpm for 10 min. The supernatant was stored at 4 °C until used for various assays. The serum glucose was estimated by the method of Freund et al.^33^, insulin^34^, aspartate aminotransferase (AST) and alanine aminotransferase (ALT)^35^, alkaline phosphatase (ALP)^36^, total protein^37^, albumin^37^, triglycerides (TG)^38^, total cholesterol^39^, creatinine^40^, urea^41^, uric acid^41^, glutathione reduced (GSH)^42^, catalase (CAT)^43^, nitric oxide (NO)^44^, malondialdehyde (MDA)^45^, Glutathione S-transferase (GST)^46^, superoxide dismutase (SOD)^47^ using Biodiagnostic kits.

### Light microscopy

Sections of earthworms through body wall and intestine 1 cm (before and after clitellum) were fixed in 10% neutral buffered formalin, then dehydrated in a graded series of ethanol, immersed in xylene, and embedded in paraffin wax (58–60 °C). Paraffin blocks were sectioned to 5 µm thickness and stained with hematoxylin and eosin^48^.

### Scanning electron microscopy (SEM) examination

The earthworms were washed several times in saline, fixed in 2.5% glutaraldehyde in buffered 0.1 M sodium cacodylate, rinsed in the same buffer at pH 7.2, post-fixed in 1% osmium tetroxide and 1.25% potassium ferrocyanide (pH 7.2) for 1 h, and dehydrated in graded series of ethanol solutions (30–100%). The samples were allowed to dry at a critical point (Bomer-900) coated with gold–palladium in a Technics Hummer V and then examined with a Jeol SEM (Model JSM7610F, Jeol Ltd., Japan) in the Electron Microscopy Unit, Faculty of Agriculture, Cairo University.

### Statistical analysis

The obtained data were statistically analyzed by one-way ANOVA, followed by the Duncan test, to determine statistical significance between different groups using SPSS software package 23.0. All values were expressed as mean ± standard error (SE), and *p* < 0.05 was considered statistically significant.

## Results

### Characterization of CSB NCs

**Fig. 1:**
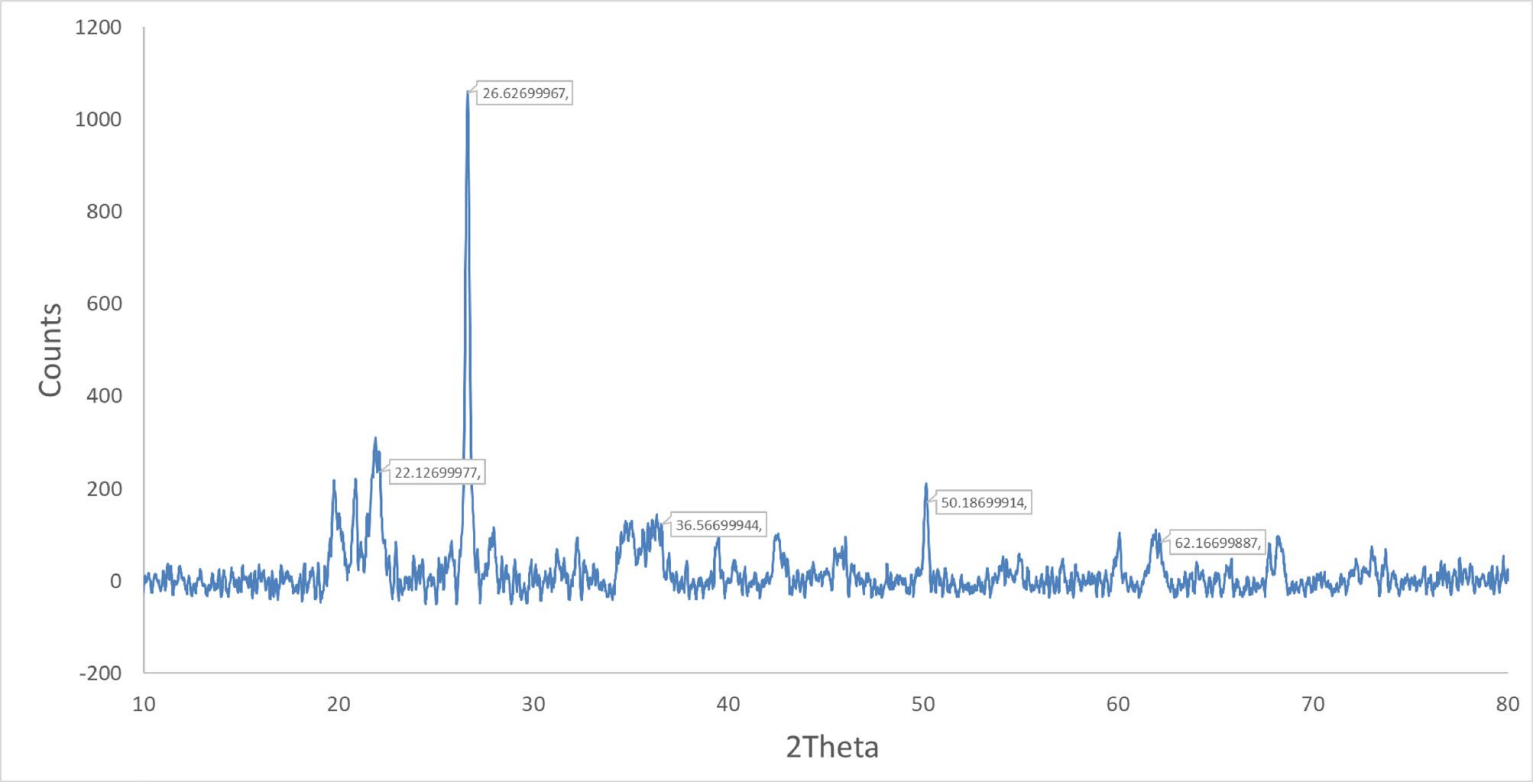
X-ray diffractometer (XRD) analysis of the synthetic CSB NCs.

X-ray diffraction (XRD) patterns of chitosan–saponin–bentonite nanocomposites (CSB NCs) illustrated that montmorillonite (M) and quartz (Q) are the main components of the used natural clay. The diffraction peaks located at 2θ were equal to 19.97°, 36.80°, and 67.13° and indexed to (020), (130), and (060) planes of montmorillonite, respectively. In addition, 2θ were equal to 26.81°, 36.25°, and 48.84° were indexed to (101), (110), and (201) planes of quartz. The other peaks are impurities corresponding to cristobalite, feldspar, and illite. The chitosan semi-crystalline broad peak at 2θ equal 22.3° (Fig. 1).

**Fig. 2:**
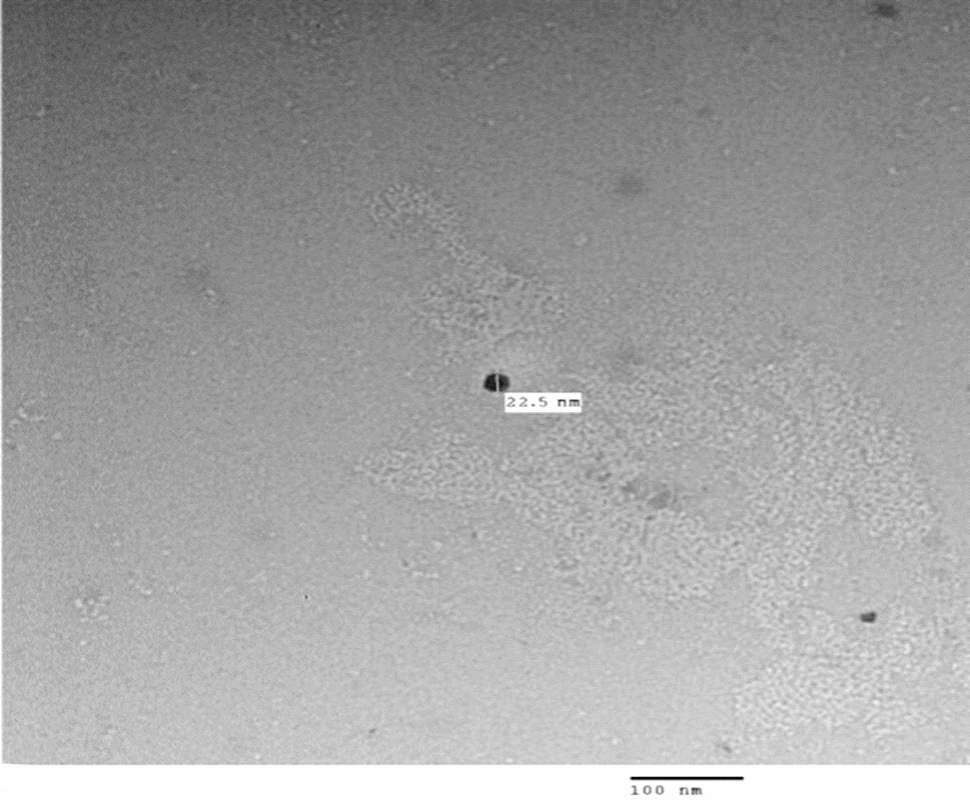
TEM micrograph of the biosynthesized CSB NCs.

Transmission electron microscopy (TEM) analysis showed that CSB NCs had an average size of 25.8 nm, spherical in shape with a smooth surface (Fig. 2).

**Fig. 3:**
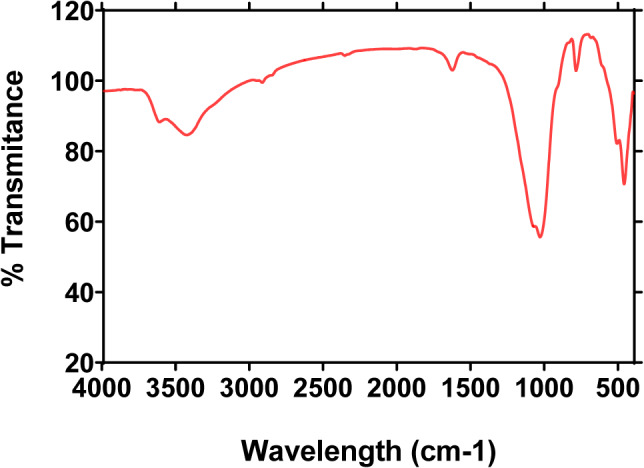
IR spectrum for the synthetic CSB NCs.

FTIR spectroscopy was used to characterize CSB NCs, the spectrum revealed peaks corresponding to their functioned group (Fig. 3), the peak at 3627 cm^−1^ indicated the –OH linkages between the octahedral and the tetrahedral layers of the silicate structure. The bentonite absorption band at 1041 cm^−1^ (asymmetrical bond stretching vibration of Si–O–Si) indicated the presence of silicate layer in the bentonite after the reaction with chitosan. The peak at 795 cm^−1^ corresponded to Si–O quartz impurity. The broadband at 3442 cm^−1^ was due to the overlapped stretching vibrations of –NH_2_ and –OH groups of chitosan. The peak at 1640 cm^−1^ is related to the vibrations of protonated amine groups chitosan chain, and the peak at 1041 cm^−1^ from the C–O group of chitosan chain.

### Evaluation of coelomocytes viability using neutral red retention time assay

The neutral red retention time dropped significantly (*p* < 0.05) to 42.78, 30.33, 22.0, and 6.33 after being exposed to the studied doses 25, 50, 100, and 150 mg of CSB NCs respectively compared to control group (Table 1).

**Table 1:** Effect of different concentrations of CSB NCs on neutral red retention time (min) of *Allolobophora caliginosa.*

CSB NCs (mg/500 g soil)	Neutral red retention time (min)	% of change
0 (Control)	53.0 ± 1.8^e^	–
25	42.78 ± 0.78^d^	− 19%
50	30.33 ± 1.27^c^	− 43%
100	22.0 ± 0.88^b^	− 58%
150	6.33 ± 0.62^a^	− 88%

Values are given as means ± SEM for 5 worms in each group. Each value not sharing a common letter superscript is significantly different (*p* < 0.05).

### Biochemical and oxidative stress assessment

The biochemical parameters showed a significant decrease (*p* < 0.05) in AST, ALT, ALP, LDH, total protein, albumin, cholesterol, triglycerides, urea, and glucose levels after exposure to the tested concentrations of CSB NCs while creatinine, uric acid, lipase, and alpha amylase levels showed a significant elevation (*p* < 0.05) as compared to the control group (Table 2).

**Table 2:** Effect of different concentrations of CSB NCs on biochemical parameters of *Allolobophora caliginosa*.

Parameters	CSB NCs (mg/500 g soil)				
	0 (Control)	25	50	100	150
ALT (U/g. tissue)	108.14 ± 2.46^d^	88.79 ± 1.78^c^	47.76 ± 4.85^b^	26.89 ± 0.9^a^	20.06 ± 1.31^a^
AST (U/g. tissue)	90.8 ± 2.12^e^	69.45 ± 2.09^d^	59.51 ± 1.01^c^	27.99 ± 1.5^b^	17..93 ± 1.83^a^
ALP (U/g. tissue)	3235.04 ± 56.74^e^	2801.15 ± 48.53^d^	1677.03 ± 61.88^c^	1363.16 ± 31.13^b^	840.57 ± 37.93^a^
Albumin (g/g. tissue)	0.25 ± 0.01^b^	0.2 ± 0.01^a^	0.2 ± 0.01^a^	0.21 ± 0.02^a,b^	0.18 ± 0.01^a^
Total proteins (g/g. tissue)	0.64 ± 0.02^e^	0.52 ± 0.02^d^	0.45 ± 0.02^c^	0.39 ± 0.01^b^	0.32 ± 0.01^a^
Alpha amylase (U/g. tissue)	0.38 ± 0.02^a^	0.52 ± 0.02^b^	0.68 ± 0.06^c^	0.79 ± 0.05^c^	1.02 ± 0.04^d^
Glucose (mg/g. tissue)	247.17 ± 6.17^d^	200.42 ± 6.46^c^	156.42 ± 3.77^b^	138.85 ± 3.49^a^	138.26 ± 4.8^a^
LDH (U/g. tissue)	39.43 ± 0.89^e^	26.7 ± 1.01^d^	23.82 ± 0.94^c^	20.56 ± 0.85^b^	13.64 ± 0.76^a^
Lipase (U/g. tissue)	9.37 ± 0.77^a^	38.01 ± 1.85^b^	43.68 ± 1.76^c^	57.29 ± 1.28^d^	65.4 ± 2.26^e^
Cholesterol (mg/g. tissue)	29.5 ± 0.72^d^	21.08 ± 1.12^c^	17.07 ± 0.38^b^	16.83 ± 0.42^b^	11.7 ± 0.4^a^
Triglycerides (mg/g. tissue)	8.32 ± 0.32^d^	5.31 ± 0.41^c^	3.72 ± 0.15^b^	3.2 ± 0.17^a,b^	2.47 ± 0.14^a^
Creatinine (mg/g. tissue)	0.52 ± 0.02^a^	0.59 ± 0.03^a^	0.67 ± 0.01^b^	0.86 ± 0.03^c^	1.01 ± 0.03^d^
Urea (mg/g. tissue)	16.94 ± 1.49^d^	12.45 ± 0.68^c^	7.69 ± 0.27^b^	5.41 ± 0.45^a^	4.61 ± 0.18^a^
Uric acid (mg/g. tissue)	1.46 ± 0.06^a^	1.6 ± 0.08^a^	2.07 ± 0.08^b^	2.36 ± 0.04^c^	2.87 ± 0.15^d^

Values are given as means ± SEM for 10 worms in each group. Each value not sharing a common letter superscript is significantly different (*p* < 0.05).

The oxidative stress markers showed a significant increase (*p* < 0.05) in MDA concentration in worms treated with CSB NCs and a significant decrease (*p* < 0.05) in the levels of NO, GSH, and GST, CAT, SOD (Table 3).

**Table 3:** Effect of different concentrations of CSB NCs on oxidative stress parameters of *Allolobophora caliginosa*.

Parameters	CSB NCs (concentration (mg/500 g soil)				
	0 (Control)	25	50	100	150
MDA (nM/g.tissue)	0.69 ± 0.02^a^	0.76 ± 0.02^a,b^	0.83 ± 0.03^b^	0.93 ± 0.03^c^	1.28 ± 0.04^d^
NO (µM/g.tissue)	40.55 ± 1.17^d^	32.81 ± 0.89^c^	30.85 ± 0.75^c^	28.39 ± 0.71^b^	22.77 ± 0.57^a^
GSH (mM/g.tissue)	1.05 ± 0.04^e^	0.83 ± 0.03^d^	0.48 ± 0.02^c^	0.29 ± 0.01^b^	0.2 ± 0.02^a^
GST (µM/g.tissue/min)	32.94 ± 1.34^e^	27.82 ± 0.83^d^	21.95 ± 1.16^c^	17.09 ± 0.55^b^	12.57 ± 0.46^a^
CAT (U/min/g.tissue)	9.21 ± 0.94^c^	8.06 ± 0.17^b,c^	7.05 ± 0.2^b^	5.21 ± 0.22^a^	4.47 ± 0.14^a^
SOD (U/g.tissue)	6.34 ± 0.16^e^	4.24 ± 0.08^d^	2.83 ± 0.11^c^	2.33 ± 0.09^b^	1.47 ± 0.1^a^

Values are given as means ± SEM for 10 worms in each group. Each value not sharing a common letter superscript is significantly different (*p* < 0.05).

### Light microscopic examination of the body wall and intestine of *A. caliginosa*

#### Body wall region before clitellum

**Fig. 4:**
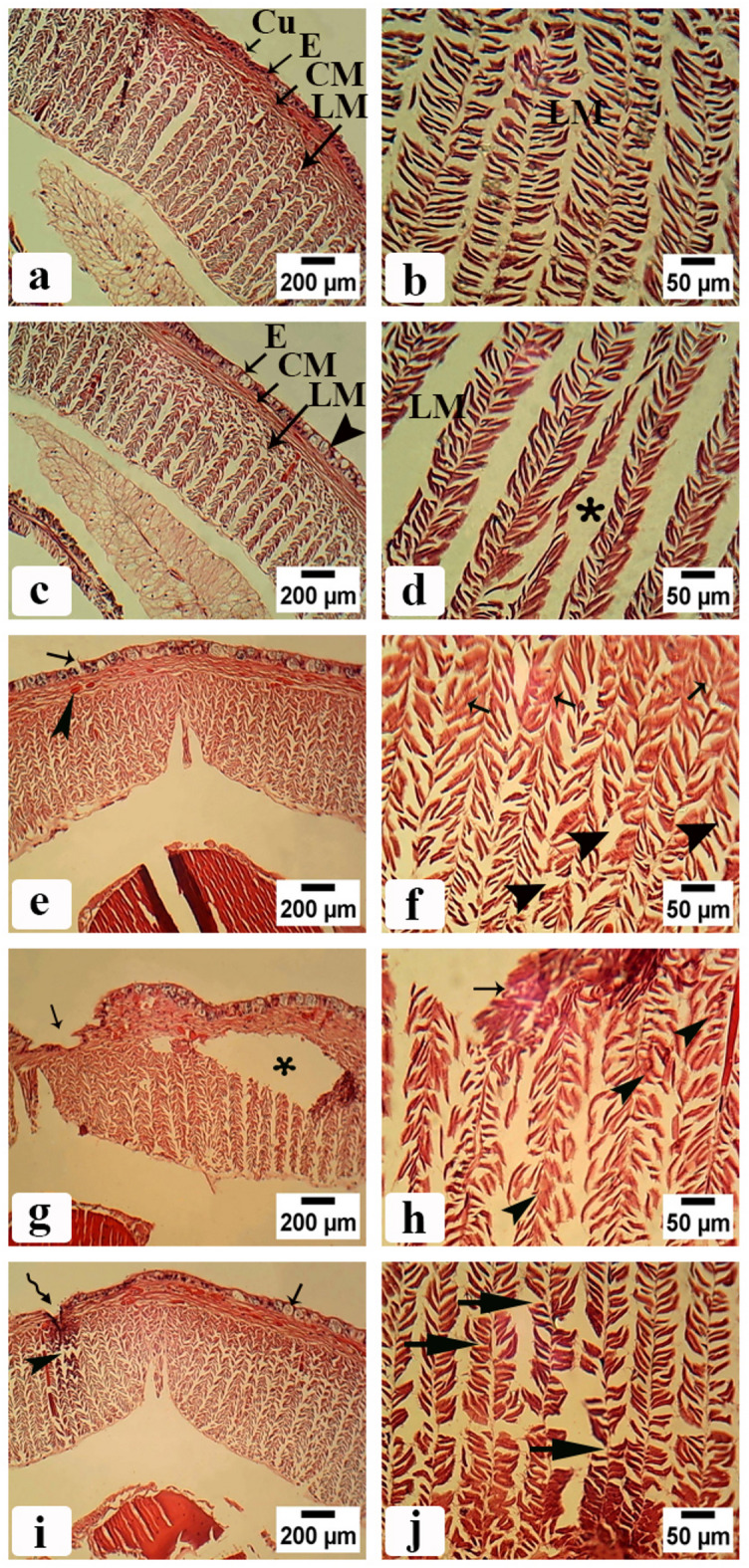
Photomicrographs of *A. caliginosa* transverse sections of body wall before clitellum (H&E). (**a**) Control group showing normal body wall structure with cuticle (Cu), epidermal cells (E), circular muscle layer (CM), longitudinal muscle layer (LM). Scale bar = 200 µm. (**b**) Magnified part of untreated earthworms body wall showing well organized longitudinal muscle fibers (LM). Scale bar = 50 µm. (**c**) Body wall of earthworms group treated with 25 mg CSB NCs/500 g soil. Scale bar = 200 µm. (**d**) High magnification of longitudinal muscle layer of the same group. Scale bar = 50 µm (asterisk: large intercellular spaces of longitudinal muscle). (**e**) Body wall of earthworms group treated with 50 mg CSB NCs/500 g soil. Scale bar = 200 µm (small arrow: enlarged cell in circular muscle layer (arrowhead)). (**f**) High magnification of longitudinal muscle layer of the same group. Scale bar = 50 µm (arrowheads: ruptured longitudinal muscle fibers and arrows: fibrotic longitudinal muscle fibers). (**g**) Body wall of earthworms group treated with 100 mg CSB NCs/500 g soil. Scale bar = 200 µm (arrow: erosion in the epidermis, asterisk: atrophy in longitudinal muscle fibers and separation between circular and longitudinal muscle layers creating a space between them). (**h**) High magnification of longitudinal muscle layer of the same group. Scale bar = 50 µm (arrow: irregular appearance of longitudinal muscle fibers, arrowheads necrosis). (**i**) Body wall of earthworms group treated with 150 mg CSB NCs/500 g soil. Scale bar = 200 µm (zigzag arrow: appearance of furrowin the epidermis, arrowhead: disrupted muscle layer, arrow: vacuolated epidermal cells with multiple nuclei). (**j**) High magnification of longitudinal muscle layer of the same group. Scale bar = 50 µm (arrows: contracted muscle fiber with atrophied muscle filaments).

As shown in Fig. 4a and b control groups had an intact body structure with normal body walls without any damage. In addition, it had compact cells and normal nuclei. The body wall consists of a smooth cuticle (Cu) with an outer epidermis filled with epidermal cells (E) with nuclei and inner muscular layers consists of circular muscle layer (CM) and longitudinal muscle layer (LM) distributed at equal intervals and well organized. Figure 4c–f showed the body wall of earthworms treated with CSB NCs 25 and 50 mg/500 g soil. The epidermal layer had been enlarged and ruptured in many regions with many vesicles. The longitudinal muscle layer had reduced in thickness and had many intercellular spaces. While the circular muscle layer had enlarged cell and fibrotic areas.

Figure 4g–j showed the body wall of earthworms treated with CSB NCs 100 and 150 mg/500 g soil. The epidermal layer had many erosions, vacuolated epidermal cells with multiple nuclei, and many furrows. The longitudinal muscle layer had atrophy, necrosis, and contracted muscle fiber with atrophied muscle filaments. While the circular muscle layer had separated from longitudinal muscle layers creating a space between them.

#### Body wall region after clitellum

**Fig. 5:**
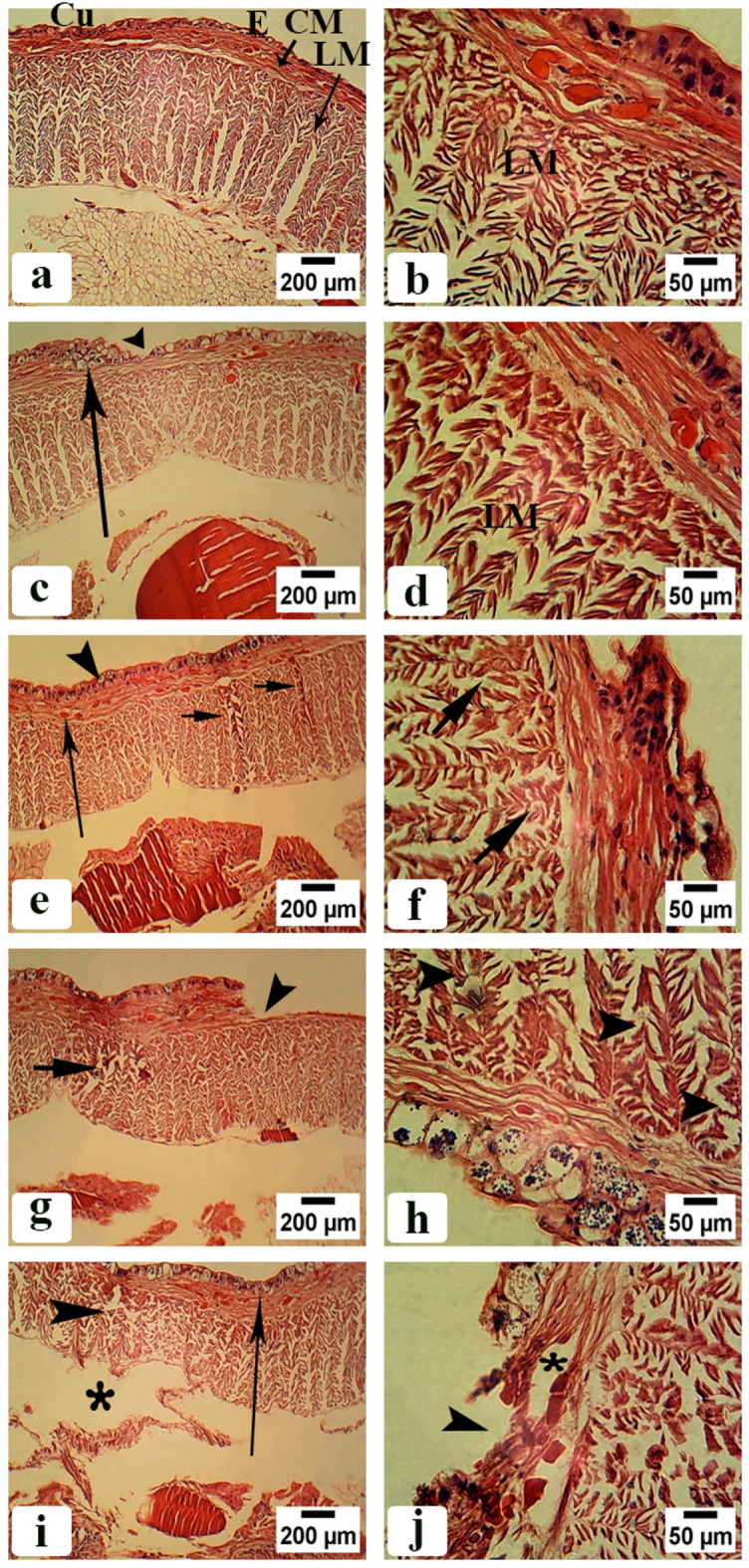
Photomicrographs of *A. caliginosa* transverse sections of body wall after clitellum (H&E). (**a**) Control group showing normal body wall structure with cuticle (Cu), epidermal cells (E), circular muscle layer (CM), longitudinal muscle layer (LM). Scale bar = 200 µm. (**b**) Magnified part of untreated earthworms body wall showing well organized longitudinal muscle fibers (LM). Scale bar = 50 µm. (**c**) Body wall of earthworms group treated with 25 mg CSB NCs/500 g soil. Scale bar = 200 µm (arrowhead: erosion in the epidermis, arrow: enlargement in the thickness of epidermal layer and abundance of vesicles). (**d**) High magnification of longitudinal muscle layer of the same group. Scale bar = 50 µm (**e**) Body wall of earthworms group treated with 50 mg CSB NCs/500 g soil. Scale bar = 200 µm (arrowhead: enlargement in the thickness of epidermal layer and abundance of vesicles, arrow: enlarged cell in circular muscle layer, smallarrows: ruptured longitudinal muscle fibers). (**f**) High magnification of longitudinal muscle layer of the same group. Scale bar = 50 µm (arrows: ruptured longitudinal muscle fibers). (**g**) Body wall of earthworms group treated with 100 mg CSB NCs/500 g soil. Scale bar = 200 µm (arrowhead: erosion in the epidermis, arrow: ruptured longitudinal muscle fibers). (**h**) High magnification of longitudinal muscle layer of the same group. Scale bar = 50 µm (arrowheads: ruptured and irregular appearance of longitudinal muscle fibers). (**i**) Body wall of earthworms group treated with 150 mg CSB NCs/500 g soil. Scale bar = 200 µm (asterisk: atrophy in longitudinal muscle fibers creating a space between its fibers, arrowhead: disrupted muscle layer, thin arrow: vacuolated epidermal cells with multiple nuclei). (**j**) High magnification of longitudinal muscle layer of the same group. Scale bar = 50 µm (asterisk: atrophy in circular muscle fibers creating a space between its fibers, arrowhead: erosion in the epidermis).

Figure 5a and b illustrated that the control groups’ bodies were structurally intact, with typical, undamaged walls. It also had normal nuclei and compact cells. The body wall was composed of a smooth cuticle (Cu), an outer layer of epidermis consisting of nucleated epidermal cells (E), and well-organized, evenly spaced inner muscular layers made up of longitudinal muscle layer (LM) and circular muscle layer (CM). Figure 5c–f indicated the body wall of earthworms treated with CSB NCs 25 and 50 mg/500 g soil. The epidermal layer had been enlarged in thickness with erotic areas and abundance of vesicles. The longitudinal muscle layer had reduced in thickness with ruptured fibers. While the circular muscle layer had enlarged cells. Figure 5g–j indicated the body wall of earthworms treated with CSB NCs 100 and 150 mg/500 g soil. The epidermal layer had vacuolated epidermal cells with multiple nuclei. The longitudinal and circular muscle layers had ruptured and atrophied muscle fibers.

#### Intestine region before clitellum

**Fig. 6:**
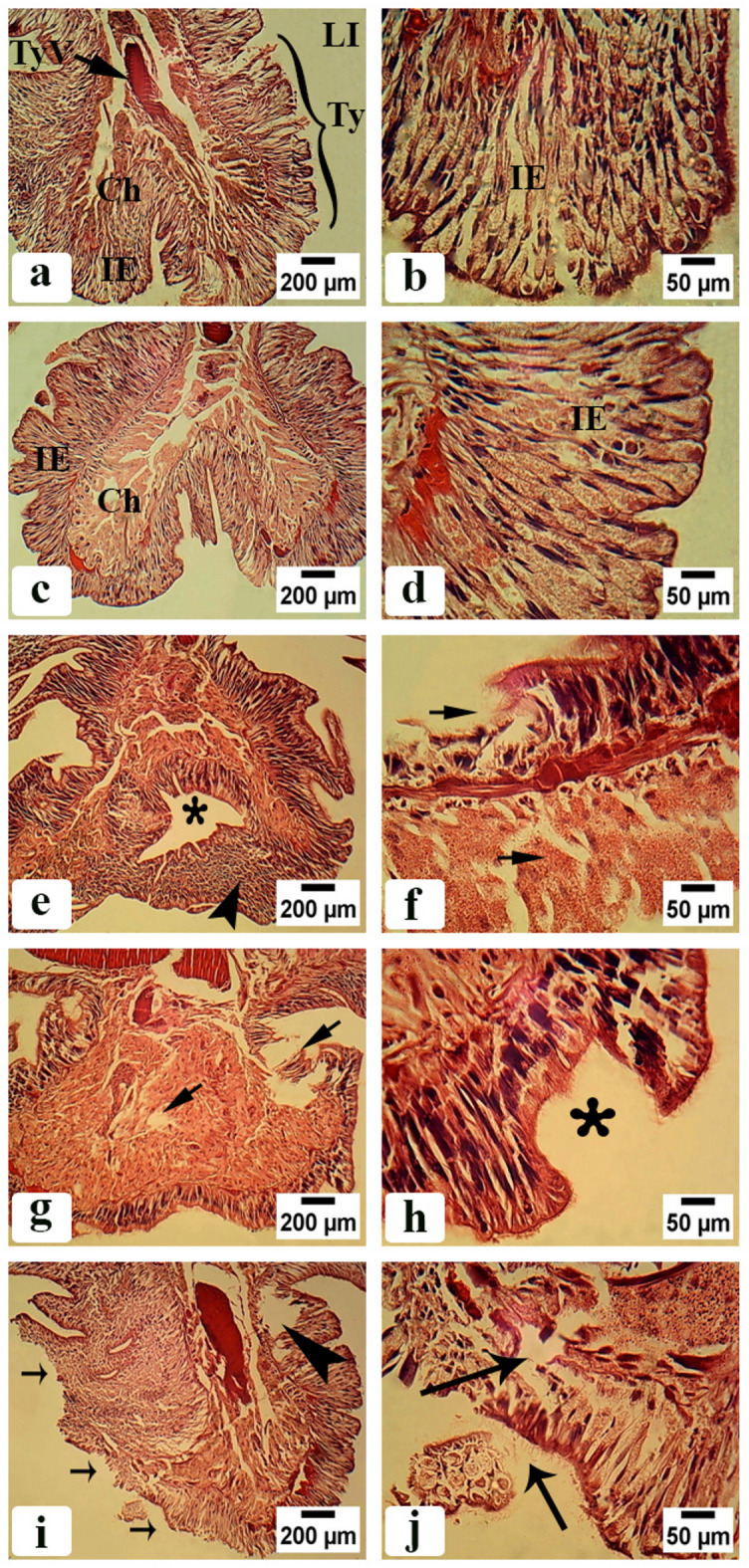
Photomicrographs of *A. caliginosa* transverse sections of intestine before clitellum (H&E). (**a**) Control group showing normal body cavity with typhlosole (Ty), chloragogen tissue (Ch), intestinal epithelium (IE), lumen of intestine (LI), typhlosole vessel (TyV). Scale bar = 200 µm. (**b**) Magnified part of untreated earthworms body cavity showing well organized intestinal epithelium (IE). Scale bar = 50 µm. (**c**) Intestine of earthworms group treated with 25 mg CSB NCs/500 g soil showing organized intestinal epithelium (IE) and chloragogen tissue (Ch). Scale bar = 200 µm. (**d**) High magnification of intestinal epithelium of the same group. Scale bar = 50 µm. (**e**) Intestine of earthworms group treated with 50 mg CSB NCs/500 g soil. Scale bar = 200 µm (asterisk: atrophy in chloragogen tissue and separation between chloragogen tissue and intestinal epithelium creating a space between them, arrowhead: formation of aggregate cell clusters in intestinal epithelium). (**f**) High magnification of intestinal epithelium of the same group. Scale bar = 50 µm (arrows: fibrotic intestinal epithelium). (**g**) Intestine of earthworms group treated with 100 mg CSB NCs/500 g soil. Scale bar = 200 µm (arrows: destruction in typhlosole tissue). (**h**) High magnification of intestinal epithelium of the same group. Scale bar = 50 µm (asterisk: necrosis of typhlosole tissue). (**i**) Intestine of earthworms group treated with 150 mg CSB NCs/500 g soil. Scale bar = 200 µm (arrows: complete destruction of gut layers, arrowhead: tissue necrosis). (**j**) High magnification of intestinal epithelium of the same group. Scale bar = 50 µm (arrows: scattered tears in the different tissue).

As shown in Fig. 6a and b control groups exhibited a normal body cavity with typhlosole (Ty), which is an internal fold, and typhlosole vessel (TyV) is in its center; its function is to increase intestine surface area for more efficient absorption of digested nutrients. Chloragogen tissue (Ch) in the intestinal wall of annelids is an important center of metabolism and the synthesis of hemoglobin. Figure 6c–f indicated the intestine of earthworms treated with CSB NCs 25 and 50 mg/500 g soil. The chloragogen tissue had atrophied, creating a space between it and the intestinal epithelium. The intestinal epithelium was characterized by aggregated cell clusters and fibrosis. Figure 6g–j showed the intestine of earthworms treated with CSB NCs 100 and 150 mg/500 g soil. The typhlosole tissue is destroyed, and necrosis is present. The gut layers have been destroyed.

#### Intestine region after clitellum

**Fig. 7:**
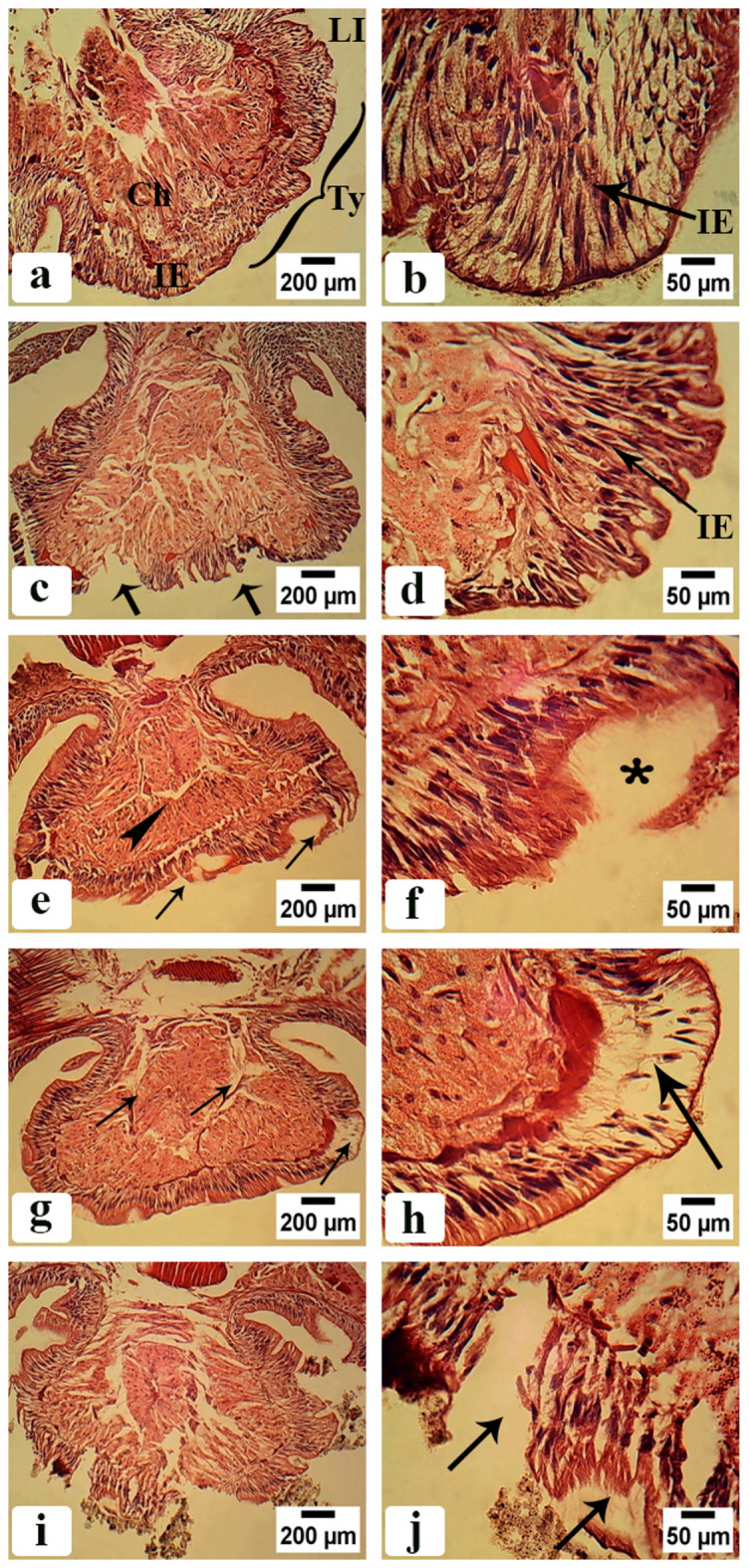
Photomicrographs of *A. caliginosa* transverse sections of intestine after clitellum (H&E). (**a**) Control group showing normal body cavity with typhlosole (Ty), chloragogen tissue (Ch), intestinal epithelium (IE), lumen of intestine (LI). Scale bar = 200 µm. (**b**) Magnified part of untreated earthworms body cavity showing well organized intestinal epithelium (IE). Scale bar = 50 µm. (**c**) Intestine of earthworms group treated with 25 mg CSB NCs/500 g soil. Scale bar = 200 µm (arrows: tissue fibrosis in intestinal epithelium). (**d**) High magnification of intestinal epithelium of the same group. Scale bar = 50 µm (**e**) Intestine of earthworms group treated with 50 mg CSB NCs/500 g soil. Scale bar = 200 µm (arrowhead: irregular appearance of chloragogen tissue, arrows: tissue fibrosis). (**f**) High magnification of intestinal epithelium of the same group. Scale bar = 50 µm (asterisk: atrophy in intestinal epithelium creating a space between its fibers), (**g**) Intestine of earthworms group treated with 100 mg CSB NCs/500 g soil. Scale bar = 200 µm (arrows: destruction in typhlosole tissue in different parts). (**h**) High magnification of intestinal epithelium of the same group. Scale bar = 50 µm (arrow: necrosis of typhlosole tissue and cavity formation like hole). (**i**) Intestine of earthworms group treated with 150 mg CSB NCs/500 g soil. Scale bar = 200 µm (**j**) High magnification of intestinal epithelium of the same group. Scale bar = 50 µm (arrows: necrosis in several parts of the tissue).

Figure 7a and b illustrates that the control groups displayed a typical body cavity with a typhlosole (Ty), an internal fold, with the typhlosole vessel (TyV) located centrally and Chloragogen tissue (Ch) is in the intestinal wall of annelids. Figure 7c–f indicated the intestine of earthworms treated with CSB NCs 25 and 50 mg/500 g soil. The tissue of the intestinal epithelium suffers from fibrosis and atrophy, resulting in gaps between its fibers. The chloragogen tissue has an irregular appearance. Figure 7g–j showed the intestine of earthworms treated with CSB NCs 100 and 150 mg/500 g soil. The typhlosole tissue is destroyed, and necrosis occurs in different parts, resulting in cavity formation, such as a hole. The gut layers are severely damaged. In multiple parts of the tissue, there is complete destruction and necrosis.

### Scanning electron microscopy (SEM) examination of *A. caliginosa*

#### The anterior part

**Fig. 8:**
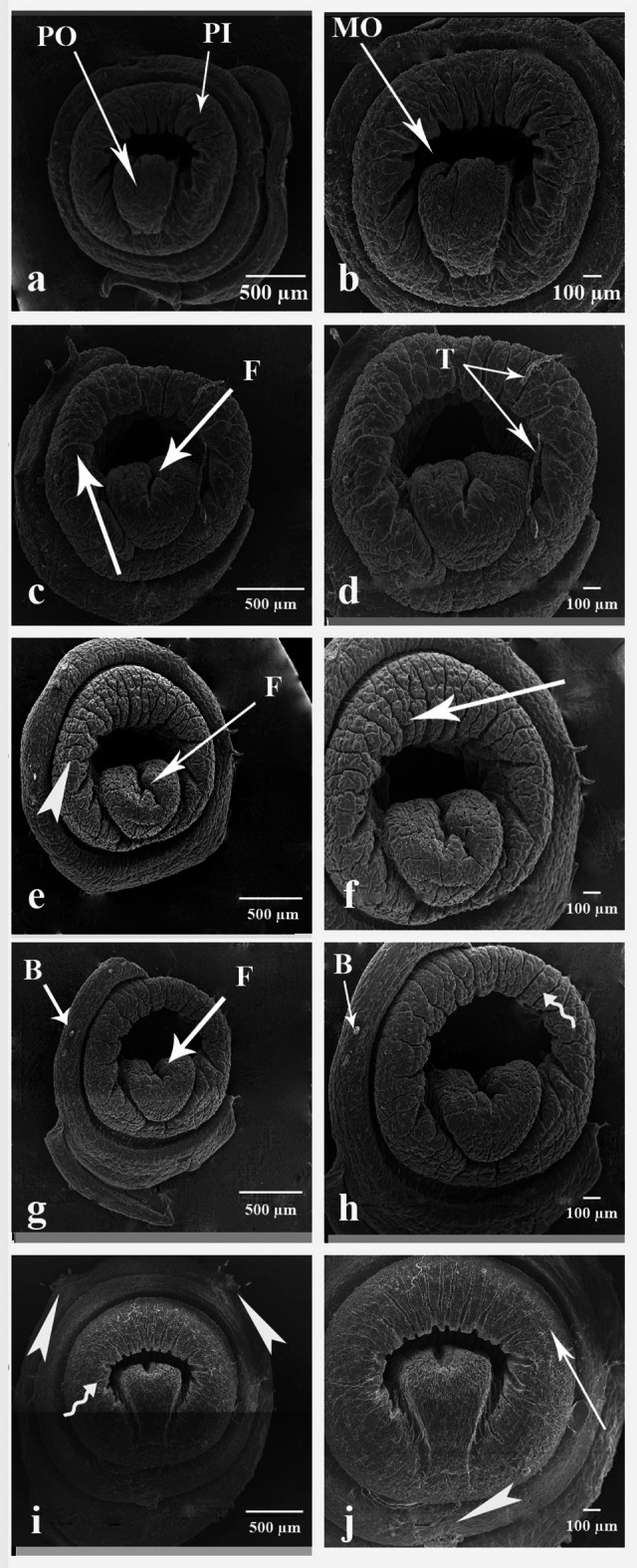
Scanning electron micrographs of the anterior part of *A. caliginosa*. (**a**) Control group showing the anterior part which consists of (MO) mouth opening, (PO) prostomium and (PI) peristomium. Scale bar = 500 µm. (**b**) Magnified part of untreated earthworm’s anterior part. Scale bar = 100 µm. (**c**) the anterior part of earthworms group treated with 25 mg CSB NCs/500 g soil. Scale bar = 500 µm (F: longitudinal fissure, arrow: multiple cracks). (**d**) High magnification of the anterior part of the same group. Scale bar = 100 µm (T: cuticle tags). (**e**) The anterior part of earthworms group treated with 50 mg CSB NCs/500 g soil. Scale bar = 500 µm (F: longitudinal fissure, arrowhead: wrinkled cuticle). (**f**) High magnification of the anterior part of the same group. Scale bar = 100 µm (arrow: multiple cracks). (**g**) The anterior part of earthworms group treated with 100 mg CSB NCs/500 g soil. Scale bar = 500 µm (B: blebs, F: longitudinal fissure. (**h**) High magnification of the anterior part of the same group. Scale bar = 100 µm (B: blebs, zigzag arro: cuticle shrinkage). (**i**) The anterior part of earthworms group treated with 150 mg CSB NCs/500 g soil. Scale bar = 500 µm (zigzag arrow: cuticle shrinkage, arrowheads: cuticle erosion). (**j**) High magnification of the anterior part of the same group. Scale bar = 100 µm (arrowhead: cuticle erosion, arrow: cuticle fibrosis).

Figure 8a and b shows scanning electron micrographs of the control group; the anterior part consists of mouth opening (MO) open in the first segment (peristomium PI), bears on its surface the prostomium (PO), a lobe overhanging the mouth. In addition, the segments appear normal and smooth. Figure 8c–f indicated the anterior part of earthworms treated with CSB NCs 25 and 50 mg/500 g soil. The tissue of the anterior part suffers from cuticle tags on some segments, longitudinal fissure on the prostomium, multiple cracks on the segment peristomium and the cuticle becoming wrinkled. Figure 8g–j indicated the anterior part of earthworms treated with CSB NCs 100 and 150 mg/500 g soil. Longitudinal fissure on the prostomium in addition to cuticle shrinkage, which becomes rougher, cuticle fibrosis, erosion in some parts, and blebs.

#### The posterior part

**Fig. 9:**
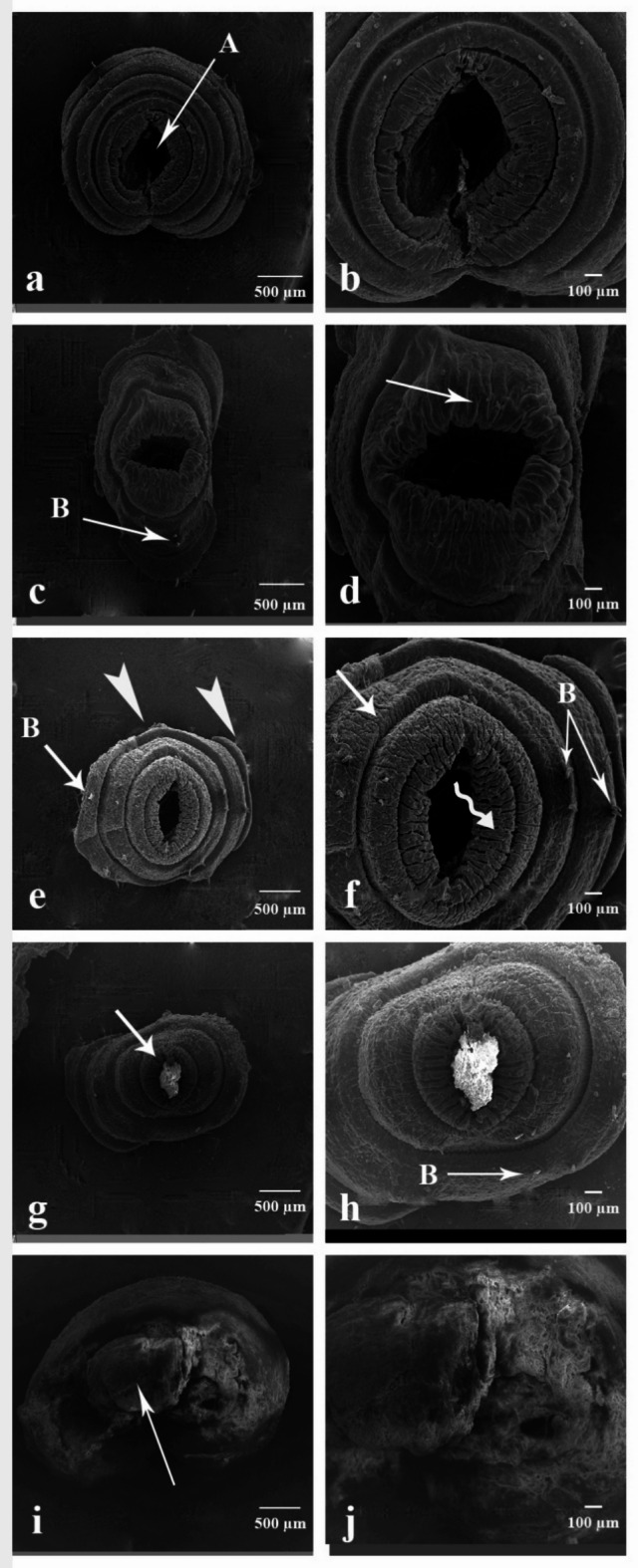
Scanning electron micrographs of the posterior part of *A. caliginosa*. (**a**) Control group showing the posterior part which consists of (A) anus. Scale bar = 500 µm. (**b**) Magnified part of untreated earthworm’s posterior part. Scale bar = 100 µm. (**c**) the posterior part of earthworms group treated with 25 mg CSB NCs/500 g soil. Scale bar = 500 µm (B: blebs). (**d**) High magnification of the posterior part of the same group. Scale bar = 100 µm (arrow: cuticle fibrosis). (**e**) The posterior part of earthworms group treated with 50 mg CSB NCs/500 g soil. Scale bar = 500 µm (B: blebs, arrowheads: cuticle erosion). (**f**) High magnification of the posterior part of the same group. Scale bar = 100 µm (arrow: cuticle fibrosis, B: blebs, zigzag arrow: cuticle shrinkage). (**g**) The posterior part of earthworms group treated with 100 mg CSB NCs/500 g soil. Scale bar = 500 µm (arrow: coelomic fluid emerged as well as the blood surrounding the wound appearance. (**h**) High magnification of the posterior part of the same group. Scale bar = 100 µm (B: blebs). (**i**) The posterior part of earthworms group treated with 150 mg CSB NCs/500 g soil. Scale bar = 500 µm (arrow: severe degradation). (**j**) High magnification of the posterior part of the same group. Scale bar = 100 µm.

Figure 9a and b shows scanning electron micrographs of the control group, the posterior part consists of the anus (A) in the last segment of the earthworm’s body. Segments seemed normal in shape, which is regular and smooth. Figure 9c–f indicated the posterior part of earthworms treated with CSB NCs 25 and 50 mg/500 g soil. There were blebs on the outer layer, shrinkage in segments’ cuticle, and some parts showed fibrosis and erosion. Figure 9g–j indicated the posterior part of earthworms treated with CSB NCs 100 and 150 mg/500 g soil. The coelomic fluid emerged as well as the blood surrounding the wound appearance, some blebs appear, the cuticle becomes wrinkled, total damage of the posterior end, and severe degradation hide all features of this part was observed.

#### The dorsal side

**Fig. 10:**
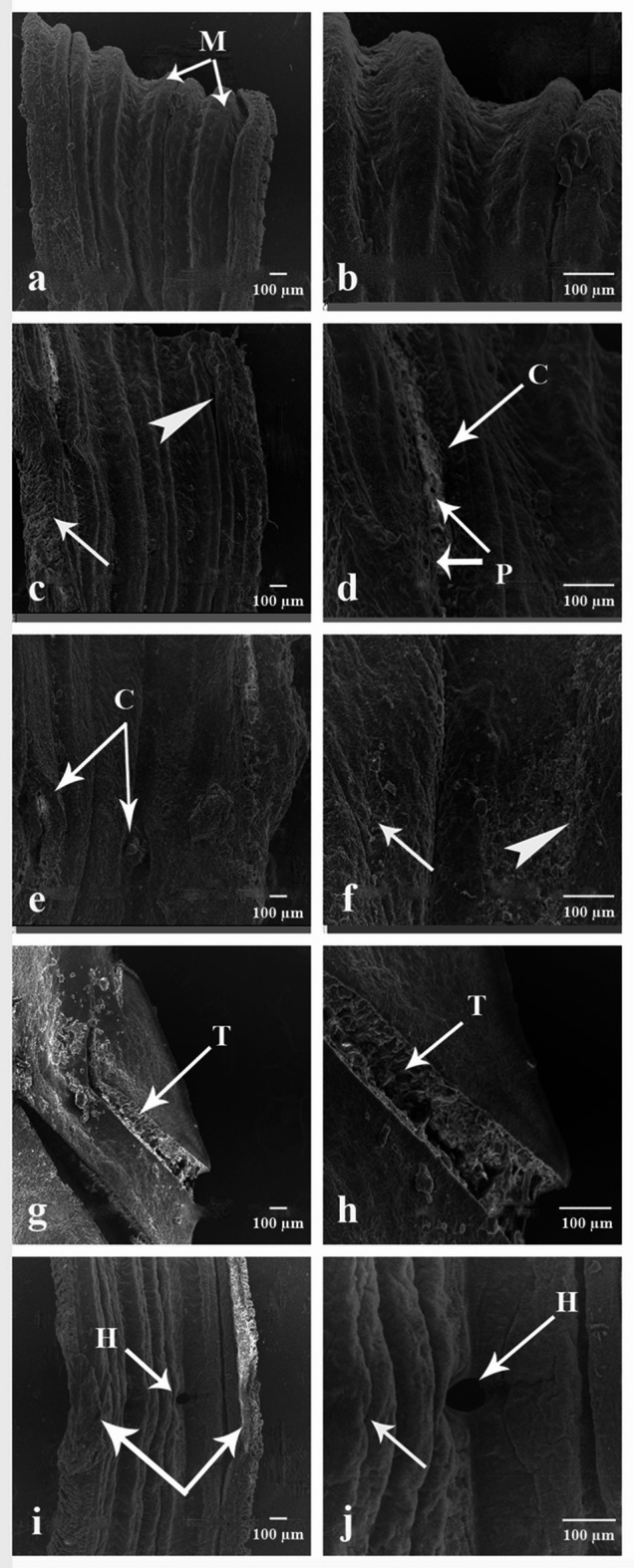
Scanning electron micrographs of the dorsal side of *A. caliginosa*. (**a**) Control group showing the dorsal side which consists of (M) metameres. Scale bar = 100 µm. (**b**) Magnified part of untreated earthworm’s dorsal side. Scale bar = 100 µm. (**c**) the dorsal side of earthworms group treated with 25 mg CSB NCs/500 g soil. Scale bar = 100 µm (arrow: cuticle fibrosis, arrowhead: cuticle has become rougher and more wrinkled). (**d**) High magnification of the dorsal side of the same group. Scale bar = 100 µm (P: pores, C: cracks). (**e**) The dorsal side of earthworms group treated with 50 mg CSB NCs/500 g soil. Scale bar = 100 µm (C: cracks). (**f**) High magnification of the dorsal side of the same group. Scale bar = 100 µm (arrow cuticle fibrosis, arrowhead: cuticle has become rougher). (**g**) The dorsal side of earthworms group treated with 100 mg CSB NCs/500 g soil. Scale bar = 100 µm (T: tear). (**h**) High magnification of the dorsal side of the same group. Scale bar = 100 µm (T: tear). (**i**) The dorsal side of earthworms group treated with 150 mg CSB NCs/500 g soil. Scale bar = 100 µm (arrows: cuticle fibrosis), H: broad hole). (**j**) High magnification of the dorsal side of the same group. Scale bar = 100 µm (arrow: cuticle fibrosis (), H: broad hole.

The dorsal side is the top of the earthworm running from the anterior to the posterior. Scanning electron micrographs of the control group show that the earthworm is divided externally into segments along the length of the body by intersegmental grooves, which form metameres (M) that are regular, smooth and parallel to each other (Fig. 10a,b). Figure 10c–f indicated the dorsal side of earthworms treated with CSB NCs 25 and 50 mg/500 g soil. Metameres have lost their regularity, and the cuticle has become rougher and more wrinkled. There were some small pores, cuticle fibrosis in multiple parts, and some cracks. Figure 10g–j indicated the dorsal side of earthworms treated with CSB NCs 100 and 150 mg/500 g soil. Tears in the cuticle, broad hole, and cuticle fibrosis.

#### The ventral side

**Fig. 11:**
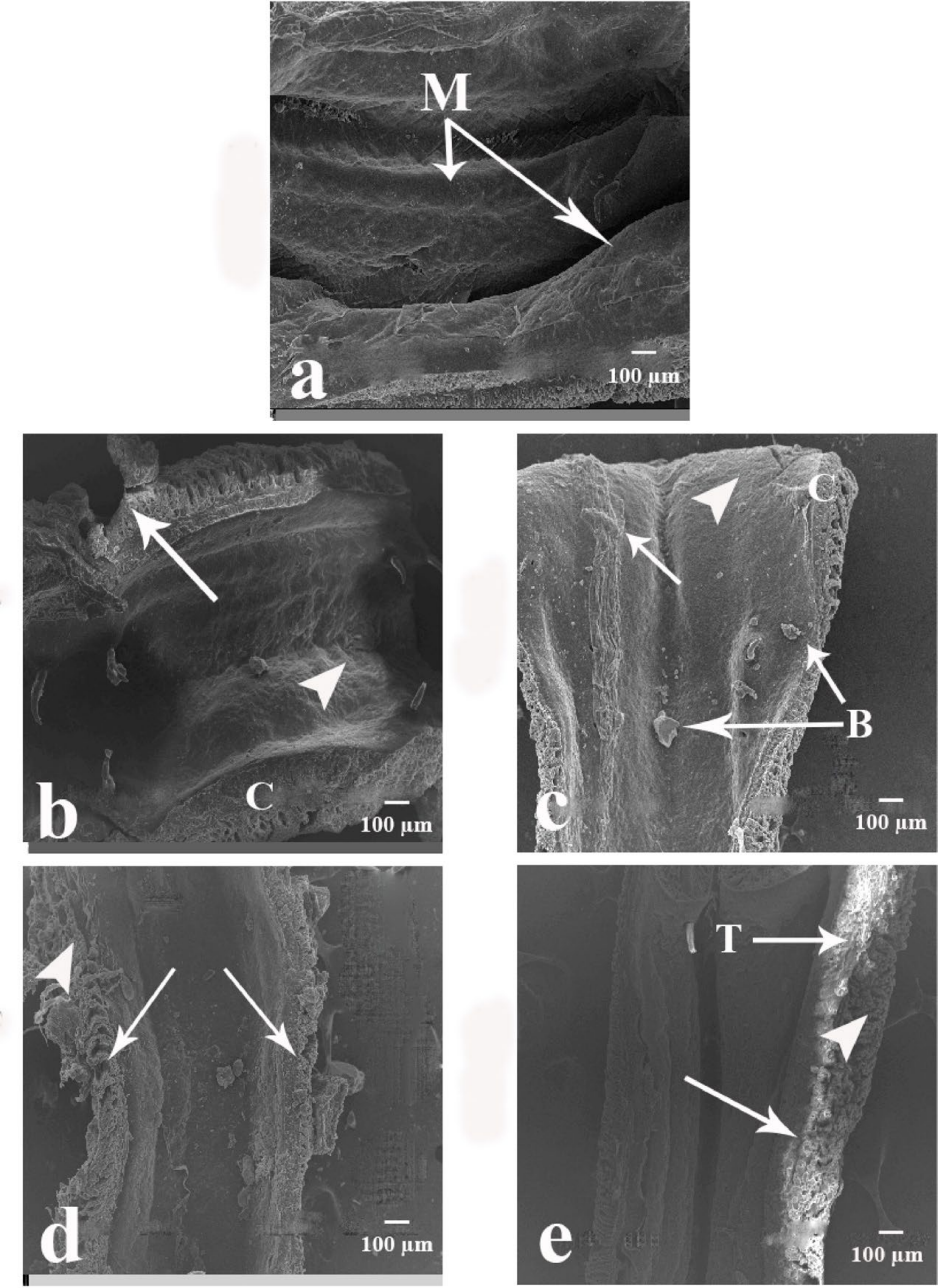
Scanning electron micrographs of the ventral side of *A. caliginosa*. (**a**) Control group showing the ventral side which consists of (M) metameres. Scale bar = 100 µm. (**b**) the ventral side of earthworms group treated with 25 mg CSB NCs/500 g soil. Scale bar = 100 µm. (arrow: cuticle fibrosis, arrowhead: cuticle has become wrinkled, C: cracks). (**c**) The ventral side of earthworms group treated with 50 mg CSB NCs/500 g soil. Scale bar = 100 µm (arrow: cuticle fibrosis, arrowhead: cuticle has become wrinkled, C: cracks, B: blebs. (**d**) The ventral side of earthworms group treated with 100 mg CSB NCs/500 g soil. Scale bar = 100 µm (arrows: damaged cuticle, arrowhead: severe erosion). (**e**) The ventral side of earthworms group treated with 150 mg CSB NCs/500 g soil. Scale bar = 100 µm (arrow: cuticle fibrosis, arrowhead: severe erosion, T: tear.

The Ventral side is the underside of the earthworm running from the anterior to the posterior. Scanning electron micrographs of the control group show that the body is divided externally into segments along the length of the body by intersegmental grooves, which form metameres (M) that are regular, smooth and parallel to each other (Fig. 11a). Figure 11b and c indicated the ventral side of earthworms treated with CSB NCs 25 and 50 mg/500 g soil. There was no longer any regularity in terms of the wrinkled cuticle. There was fibrosis of the cuticle in various areas, cracks and blebs. Figure 11d and e indicated the ventral side of earthworms treated with CSB NCs 100 and 150 mg/500 g soil. There were tears, damage in multiple parts, fibrosis and severe erosion in the cuticle.

## Discussion

The neutral red retention time assay is useful for detecting decreased lysosomal membrane stability in cells, which is frequently related to exposure to environmental contaminants, including nanomaterials^32^. The current study revealed a significant decrease in neutral red retention time (min) with increased concentration of CSB NC. The neutral red retention dye is exclusively absorbed and stored by lysosomes in healthy cells. The dye is shown to permeate from the lysosomes into the cytoplasm, signifying damage and instability of the lysosomal membranes^49^.

Biochemical parameters are sensitive indices to changes due to xenobiotics and can constitute an important diagnostic tool in toxicological studies^50^. The aminotransferases (AST and ALT) catalyze the interconversion of amino acids and α-ketoacids by the transfer of amino groups^51^. The depletion of the transaminases in the present study seems necessary for the animal to restore the amino acid balance since transaminases are necessary in regulating the concentrations of keto and amino acids^52^.

Multifunctional alkaline phosphatases (ALP) are widely known as established biomarkers for evaluating environmental stress, which catalyzes phosphomonoester substrates at a pH of 9.8 in an alkaline medium^53^. In addition, these enzymes play a crucial role in various functions, including protein synthesis and humus formation^54^. The reduction of ALP activity in the current study may be related to the interruption of protein synthesis because of the toxin on the general metabolism of the animal^55^.

Lactate dehydrogenase (LDH) is a crucial metabolic enzyme. Its activity variations have been investigated in several soil invertebrates in response to changes in their environment^56^. LDH plays a fundamental role in energy production as it facilitates the conversion of pyruvate to lactate during anaerobic glycolysis^57^. A decrease in LDH activity in the present study indicates a disturbance in energy metabolism in the exposed earthworm to nanocomposite^58^.

Glucose is an essential nutrient for cellular metabolism. It is predominantly retained in the tissues of earthworms as glycogen, a polysaccharide that serves an essential storage function^59^. Within the present investigation, a reduction in glucose concentration was observed across all groups under examination. This could be attributed to the fact that worms require glucose metabolism for energy production and environmental resistance^60^.

Proteins are the major constituents of earthworm body walls, assuming structural and energy storage functions when glycogen levels are low^61^. In the current study, there was a decrease in total protein and albumin concentrations in the examined earthworm. In agreement with our results, the animals in unfavorable environments experiencing a drop in glucose content may shift to protein metabolism to supply needed energy^60^.

Earthworms are ammoniotelic and ureotelic organisms. Their nitrogenous waste contains urea, water, traces of ammonia, and creatinine, which nephridia eliminate^62,63^. Each earthworm nephridium is like a mammalian kidney nephron. Earthworm nephrostome functions resemble the mammalian glomerulus. The first and second loops resemble the proximal and distal tubules of the mammalian kidney, which reside in the cortex^64^. The current investigation showed that earthworms consuming CSB NC for 7 days caused a decrease in urea and an increase in creatinine and uric acid concentrations. In agreement with our results, the reduction of nephrostome filtration rate following treatment with CSB NCs may be attributed to nephridial loop blockage induced after apoptosis and cellular necrosis^65^.

Lipids are the most important long-term energy reserves in organisms. Phospholipids, cholesterol and triglycerides are the main lipids found in all invertebrates^66^. Cholesterol plays a crucial role in the membrane structure and is also vital for other signaling mechanisms^67^. It serves as a precursor for significant categories of biologically active substances, such as steroid hormones, oxysterols, vitamin D, and bile acids^68^. Concerning the effect of CSB NCs, the obtained results showed a significant increase in the levels of cholesterol and triglycerides in earthworms. In agreement with the report of Nandurkar and Zambare^69^, the increase in total cholesterol and triglycerides levels may be due to the stressful conditions to which the organisms have encountered.

Earthworms contain abundant enzymes (amylase, cellulase, and lipase) useful for the bioremediation of contaminated soils, in animal feeds for improving the digestion of proteins, lipids, and carbohydrates, and in the saccharification of starch, detergents, animal and human therapeutics^70^. Lipase in earthworms is the primary digestant that breaks down oils and fats into smaller molecules^71^. Amylase can hydrolyze the starch molecules into polymers composed of glucose units^72^. In the current investigation, incubating the earthworm in CSB NCs showed a significant increase in the activities of lipase and amylase enzymes, indicating the high impairment of earthworm digestive capacity. In addition, elevated lipase could be attributed to increased lipid mobilization and reduced lipid transport away from certain bodily regions^73^.

One of the potential mechanisms for the toxicity of CSB NCs in the earthworm is oxidative stress (OXS), which is a suitable parameter. It has been hypothesized that the scavenging capacity of any organism is surpassed by ROS when exposed to xenobiotic compounds^74,75^. The severity of stress and the physical and chemical conditions within the cell determine the repercussions of ROS formation^76^. Malondialdehyde (MDA) is one of the extremely reactive metabolic byproducts that are generated when free oxygen radicals combine with lipid peroxide in tissues^77,78^. The results of the present investigation showed an elevation of MDA content in the earthworm after exposure to CSB NCs that may be due to the anti-oxidative system failing to conquer the stress caused by ROS, leading to the increment of MDA content^31,79^.

Antioxidants are substances that counteract and deactivate the harmful effects of free radicals, safeguarding against oxidative stress and cell damage^80,81^. The endogenous antioxidants include GSH, CAT, GST, glutathione peroxidase, and thioredoxin, while exogenous sources refer to important nutrients such as vitamin C, vitamin E, beta-carotene, selenium, bioflavonoids, proanthocyanidins, and N-acetyl cysteine (NAC)^82^. The present investigation showed a depletion in the GSH content, CAT, and GST activities of earthworm exposed to CSB NCs. The reduction in GSH content, CAT, and GST activities might be due to the enrichment of the peroxidation end product, MDA, which is known to inhibit protein synthesis and the activities of certain enzymes^83,84^. Furthermore, dropped endogenous antioxidants in treated earthworms may be related to a reduced capability to neutralize reactive oxygen species and increased susceptibility to oxidative stress^85^. Nitric oxide (NO) is regarded as a crucial signaling molecule involved in a variety of physiological functions, such as immune system function, vascular control, nervous system transmission, and the etiology of various disorders^86^. The presence of NO is well demonstrated in all vertebrates. NO is an effective chain-breaking antioxidant in free radical-mediated lipid oxidation (LPO). It reacts rapidly with peroxyl radicals as a sacrificial chain-terminating antioxidant^87^. Concerning the effect of CSB NCs, the obtained results showed a significant decrease in the level of NO in the treated earthworm. The reduction in the NO content after exposure to CSB NCs indicates a condition of OXS, which may arise due to an imbalance in ROS formation and the antioxidant defense system of the cells^88^. In addition, the enhanced free-radical concentration resulting from OXS conditions can cause loss of NO antioxidant action^89^.

The examination of *Allolobophora caliginosa* by light and scanning electron microscopy is important to reveal the morphological changes. The cuticle is the tough extracellular surface, which protects the worm from the environment and maintains the morphology and integrity of the body^90^.

The results obtained with the aid of the SEM in the present study regarding the treatment of earthworms with the CSB NCs clearly showed that the surface topography of the recovered earthworms exhibited pronounced damage in the cuticle due to the presence of multiple vesicles, pores, sloughing, and blebs. This finding agrees with García-Arcos, Jha, Waterman and Piel^90^ who showed severe damage in the cuticle of the earthworm after treatment with nickel oxide nanoparticle. One of the hallmark effects of nanocomposites was the destruction of the worm’s cuticle^91^. Furthermore, nanocomposites affect the cuticle through the binding of the released positive ions to the negatively charged cell membrane, which interferes with the membrane integrity. Nanocomposite ions may also adhere to the membrane wall, causing pores and holes through which they also can penetrate inside the organism. Moreover, the bleb and vesicles represent the earthworm’s attempt to replace its damaged surface membrane in response to the action of nanocomposites^90^.

## Conclusion

The current study found that earthworm *A. caliginosa* can be used as a soil quality bioindicator. Exposure to CSB NC caused physiological and histological alteration in earthworms. This study emphasizes the urgent need to evaluate the environmental safety of nanocomposites used in water treatment.

## Data Availability

The datasets used and/or analyzed during the current study are available from the corresponding author on reasonable request.

## References

[1] Bochynska, S. et al. The impact of water pollution on the health of older people. *Maturitas* **185**, 107981 (2024). 38555759 10.1016/j.maturitas.2024.107981

[2] Rashtian, J., Chavkin, D. E. & Merhi, Z. Water and soil pollution as determinant of water and food quality/contamination and its impact on female fertility. *Reprod. Biol. Endocrinol. RB&E* **17**(1), 5 (2019). 30636624 10.1186/s12958-018-0448-5 PMC6330570

[3] Soliman, A. M., Mohamed, A. S., Abdel-Khalek, A. A. & Badran, S. R. Impact of polyvinyl chloride nano-plastics on the biochemical status of *Oreochromis niloticus* under a predicted global warming scenario. *Sci. Rep.* **15**(1), 3671 (2025). 39880882 10.1038/s41598-025-87558-8 PMC11779928

[4] Mohamed, A. F., Mohamed, A. S., Abdel-Khalek, A. A. & Badran, S. R. Synergistic impact of temperature rises and ferric oxide nanoparticles on biochemical and oxidative stress biomarkers in *Oreochromis niloticus*: Relevant environmental risk assessment under predicted global warming. *Environ. Monit. Assess.* **197**(4), 409 (2025). 40095155 10.1007/s10661-025-13789-x

[5] Münzel, T. et al. Soil and water pollution and cardiovascular disease. *Nat. Rev. Cardiol.* **22**(2), 71–89 (2025). 39317838 10.1038/s41569-024-01068-0

[6] Abdelaziz, M. H., ElRakabawi, M. S., Soliman, A. M. & Mohamed, A. S. Freshwater bivalve *Coelatura aegyptiaca* as a sensitive bioindicator for zinc oxide/ chitosan nanocomposites toxicity. *Curr. Chem. Biol.* **19**, e22127968350685 (2025).

[7] Münzel, T., Hahad, O., Daiber, A. & Landrigan, P. J. Soil and water pollution and human health: What should cardiologists worry about?. *Cardiovasc. Res.* **119**(2), 440–449 (2023). 35772469 10.1093/cvr/cvac082 PMC10064841

[8] Tripathy, J. et al. Advances in nanoparticles and nanocomposites for water and wastewater treatment: A review. *Water* **16**(11), 1481 (2024).

[9] Ali, S. B. et al. Potential protective efficacy of biogenic silver nanoparticles synthesised from earthworm extract in a septic mice model. *BMC Biotechnol.* **24**(1), 79 (2024). 39394109 10.1186/s12896-024-00901-1 PMC11468494

[10] Asztemborska, M., Jakubiak, M., Stęborowski, R., Chajduk, E. & Bystrzejewska-Piotrowska, G. Titanium dioxide nanoparticle circulation in an aquatic ecosystem. *Water Air Soil Pollut.* **229**(6), 208 (2018). 29950745 10.1007/s11270-018-3852-8 PMC5997115

[11] Abdelmawgood, I. A. et al. Chrysin-loaded poly (lactic-co-glycolic acid) nanoparticles alleviate sepsis-induced splenic injury by regulating myeloid-derived suppressor cells. *Immunol. Res.* **73**(1), 80 (2025). 40358797 10.1007/s12026-025-09634-5

[12] Ebeed, B. W. et al. β-Glucan nanoparticles alleviate acute asthma by suppressing ferroptosis and DNA damage in mice. *Apoptosis* **30**(1), 35–54 (2025). 39305381 10.1007/s10495-024-02013-9 PMC11799111

[13] Bharadwaz, A. & Jayasuriya, A. C. Recent trends in the application of widely used natural and synthetic polymer nanocomposites in bone tissue regeneration. *Mater. Sci. Eng. C Mater. Biol. Appl.* **110**, 110698 (2020). 32204012 10.1016/j.msec.2020.110698 PMC7433904

[14] Laysandra, L. et al. Highly adsorptive chitosan/saponin-bentonite composite film for removal of methyl orange and Cr(VI). *Environ. Sci. Pollut. Res. Int.* **26**(5), 5020–5037 (2019). 30600491 10.1007/s11356-018-4035-2

[15] Singh, S. & Batra, R. Nanotechnology in wastewater treatment: A review. *Novel Appl. Polym. Waste Manag.* **2018**, 173–182 (2018).

[16] Turan, N. B., Erkan, H. S., Engin, G. O. & Bilgili, M. S. Nanoparticles in the aquatic environment: Usage, properties, transformation and toxicity—A review. *Process Saf. Environ. Prot.* **130**, 238–249 (2019).

[17] Guo, X. et al. Preparation of chitosan-modified bentonite and its adsorption performance on tetracycline. *ACS Omega* **8**(22), 19455–19463 (2023). 37305296 10.1021/acsomega.3c00745 PMC10249085

[18] Shen, Q., Xu, M.-H., Wu, T., Pan, G.-X. & Tang, P.-S. Adsorption behavior of tetracycline on carboxymethyl starch grafted magnetic bentonite. *Chem. Pap.* **76**(1), 123–135 (2022).

[19] Shariatinia, Z. Pharmaceutical applications of chitosan. *Adv. Coll. Interface Sci.* **263**, 131–194 (2019). 10.1016/j.cis.2018.11.008 30530176

[20] Qutb, S. A., Soliman, A. M., Fahmy, S. R. & Mohamed, A. S. Efficacy of eugenol loaded chitosan nanoparticles on sepsis induced liver injury in rats. *Recent Adv. Inflamm. Allergy Drug Discov.*10.2174/0127722708334976241004041438 (2025). 10.2174/0127722708334976241004041438 41392915

[21] Bhatt, P., Joshi, S., Urper Bayram, G. M., Khati, P. & Simsek, H. Developments and application of chitosan-based adsorbents for wastewater treatments. *Environ. Res.* **226**, 115530 (2023). 36863653 10.1016/j.envres.2023.115530

[22] Kurniawan, A. et al. Utilization of rarasaponin natural surfactant for organo-bentonite preparation: Application for methylene blue removal from aqueous effluent. *Mesoporous Mater.* **142**(1), 184–193 (2011).

[23] Omar, T. Y. et al. Biointerfrence between zinc oxide/alginate nanocomposites and freshwater bivalve. *Biointerface Res. Appl. Chem.* **13**, 277 (2022).

[24] Rajput, V. et al. Accumulation of nanoparticles in the soi–-plant systems and their effects on human health. *Ann. Agric. Sci.* **65**(2), 137–143 (2020).

[25] Chen, H. Bioavailability, metal based nanoparticles in agricultural system: behavior, transport, and interaction with plants. *Chem. Speciat. Bioavailab.* **30**(1), 123–134 (2018).

[26] Rajput, V. et al. ZnO and CuO nanoparticles: A threat to soil organisms, plants, and human health. *Environ. Geochem. Health Inf. Sci. Syst.* **42**, 147–158 (2020). 10.1007/s10653-019-00317-3 31111333

[27] Ghai, S. & Kaur, A. Interaction of nanoparticles to soil pollutants. In *The Role of Nanoparticles in Plant Nutrition under Soil Pollution: Nanoscience in Nutrient Use Efficiency* (eds Rajput, V. D. et al.) 309–331 (Springer, 2022).

[28] Parmar, T. K., Deepak, R. & Agrawal, Y. K. Bioindicators: The natural indicator of environmental pollution. *Front. Life Sci.* **9**(2), 110–118 (2016).

[29] Ahmed, N. & Al-Mutairi, K. A. Earthworms effect on microbial population and soil fertility as well as their interaction with agriculture practices. *Sustainability* **14**(13), 7803 (2022).

[30] Navarro Pacheco, N. I. et al. In vitro interactions of TiO_2_ nanoparticles with earthworm coelomocytes: Immunotoxicity assessment. *Nanomaterials* **11**(1), 250 (2021). 33477826 10.3390/nano11010250 PMC7832855

[31] Pacheco, N. I. N. et al. Effects of silver sulfide nanoparticles on the earthworm *Eisenia andrei*. *Comp. Biochem. Physiol. C Toxicol. Pharmacol.* **257**, 109355 (2022). 35489639 10.1016/j.cbpc.2022.109355

[32] Hu, W. et al. Neutral red retention time assay in determination of toxicity of nanoparticles. *Mar. Environ. Res.* **111**, 158–161 (2015). 26065811 10.1016/j.marenvres.2015.05.007

[33] Freund, A., Johnson, S. B., Rosenbloom, A., Alexander, B. & Hansen, C. A. Subjective symptoms, blood glucose estimation, and blood glucose concentrations in adolescents with diabetes. *Diabetes Care* **9**(3), 236–243 (1986). 3731991 10.2337/diacare.9.3.236

[34] Herbert, V., Lau, K.-S., Gottlieb, C. W. & Bleicher, S. J. Coated charcoal immunoassay of insulin. *J. Clin. Endocrinol. Metab.* **25**(10), 1375–1384 (1965). 5320561 10.1210/jcem-25-10-1375

[35] Reitman, S. & Frankel, S. A colorimetric method for the determination of serum glutamic oxalacetic and glutamic pyruvic transaminases. *Am. J. Clin. Pathol.* **28**(1), 56–63 (1957). 13458125 10.1093/ajcp/28.1.56

[36] Belfield, A. & Goldberg, D. M. Revised assay for serum phenyl phosphatase activity using 4-amino-antipyrine. *Enzyme* **12**(5), 561–573 (1971). 5169852 10.1159/000459586

[37] Tietz, N. J. T. Specimen collection and processing; sources of biological variation. In *Textbook of Clinical Chemistry* 2nd edn (WB Saunders, 1994).

[38] Tietz, N., Pruden, E., Ronald, W. & Adrich, J. Lipids, lipoproteins and apolipoproteins. *Fundam. Clin. Chem.* **4**, 968–971 (2008).

[39] Allain, C. C., Poon, L. S., Chan, C. S., Richmond, W. & Fu, P. C. Enzymatic determination of total serum cholesterol. *Clin. Chem.* **20**(4), 470–475 (1974). 4818200

[40] Vella, F. Textbook of clinical chemistry. In *Biochemical Education* Vol. 14(3) (ed. Tietz, N. W.) 146–146 (W B Saunders, 1986).

[41] Tietz, N. J. A. In *Effect of Live Yeast Incorporation in Compound diet on Digestive Enzyme Activity in Sea Bass Larvae* Vol. 204 (eds Tovar, D. et al.) 113–123 (Clinical Guide to Laboratory Tests, Berlin, 2002).

[42] Beutler, E., Duron, O. & Kelly, B. M. Improved method for the determination of blood glutathione. *J. Lab. Clin. Med.* **61**, 882–888 (1963). 13967893

[43] Aebi, H. Catalase in vitro. In *Methods in Enzymology* Vol. 105 121–126 (Elsevier, 1984). 10.1016/s0076-6879(84)05016-3 6727660

[44] Montgomery, H. A. C. & Dymock, J. The determination of nitrite in water. *Analyst* **86**, 414 (1961).

[45] Ohkawa, H., Ohishi, N. & Yagi, K. Assay for lipid peroxides in animal tissues by thiobarbituric acid reaction. *Anal. Biochem.* **95**(2), 351–358 (1979). 36810 10.1016/0003-2697(79)90738-3

[46] Habig, W. H., Pabst, M. J. & Jakoby, W. B. Glutathione S-transferases. The first enzymatic step in mercapturic acid formation. *J. Biol. Chem.* **249**(22), 7130–7139 (1974). 4436300

[47] Kakkar, P., Das, B. & Viswanathan, P. N. A modified spectrophotometric assay of superoxide dismutase. *Indian J. Biochem. Biophys.* **21**(2), 130–132 (1984). 6490072

[48] Yang, X., Shang, G. & Wang, X. Biochemical, transcriptomic, gut microbiome responses and defense mechanisms of the earthworm *Eisenia fetida* to salt stress. *Ecotoxicol. Environ. Saf.* **239**, 113684 (2022). 35623149 10.1016/j.ecoenv.2022.113684

[49] Miller, M. A., Bankier, C., Al-Shaeri, M. A. M. & Hartl, M. G. J. Neutral red cytotoxicity assays for assessing in vivo carbon nanotube ecotoxicity in mussels—Comparing microscope and microplate methods. *Mar. Pollut. Bull.* **101**(2), 903–907 (2015). 26549297 10.1016/j.marpolbul.2015.10.072

[50] Vlasenko, R. P., Mezhzherin, S. V., Garbar, A. V. & Kotsuba, Y. Polyploid races, genetic structure and morphological features of earthworm *Aporrectodea rosea* (Savigny, 1826) (Oligochaeta, Lumbricidae) in Ukraine. *Comp. Cytogenet.* **5**(2), 91–103 (2011). 24260622 10.3897/compcytogen.v5i2.968 PMC3833736

[51] Beltagi, S. M. H., Al-Shinnawy, M. S., Elkattan, N.A.-R.I. & Yousef, H. N. Effects of sublethal doses of selected botanical molluscicides on oxygen consumption of the brown garden snail, *Eobania vermiculata*. *Egypt. J. Hosp. Med.* **40**(1), 402–410 (2010).

[52] Ling, Z. N. et al. Amino acid metabolism in health and disease. *Signal Transd. Target. Ther.* **8**(1), 345 (2023). 10.1038/s41392-023-01569-3 PMC10497558 37699892

[53] Mandal, S., Chakravorty, P. P. & Kundu, J. K. Relative toxicity of two selected fungicides on acid phosphatase and alkaline phosphatase activity of epigeic earthworm Eisenia Fetida (Oligochaeta). *World Wide J. Multidis. Res. Dev. WWJMRD* **4**, 14–17 (2017).

[54] Shi, Z., Wen, M., Zhang, J., Tang, Z. & Wang, C. Effect of phenanthrene on the biological characteristics of earthworm casts and their relationships with digestive and anti-oxidative systems. *Ecotoxicol. Environ. Saf.* **193**, 110359 (2020). 32097786 10.1016/j.ecoenv.2020.110359

[55] Çakır, B., Klobučar, G. & Akat Çömden, E. Investigating the toxic effects of ethoprophos on *Eisenia fetida*: Integrating light microscopy, scanning electron microscopy, and biochemical analysis. *Chemosphere* **350**, 141019 (2024). 38141679 10.1016/j.chemosphere.2023.141019

[56] Neetu Chouhan, N. C. & Tripathi, G. Effects of zinc oxide nanoparticles on some biochemical constituents of *Eisenia fetida*. *Biochem. Cell. Arch.* **9**(11), 83–89 (2019).

[57] Li, X. et al. Lactate metabolism in human health and disease. *Signal Transd. Target. Ther.* **7**(1), 305 (2022). 10.1038/s41392-022-01151-3 PMC9434547 36050306

[58] Vaidya, V. V. & Jadhav, S. S. Toxic trio: Zinc, copper, and mercury’s effects on earthworm enzymes. *Uttar Pradesh J. Zool.* **45**(6), 172–183 (2024).

[59] Zhang, Y. et al. Metabolite changes associated with earthworms (*Eisenia fetida*) graphene exposure revealed by matrix-assisted laser desorption/ionization mass spectrometry imaging. *Ecotoxicol. Environ. Saf.* **205**, 111102 (2020). 32836152 10.1016/j.ecoenv.2020.111102

[60] Li, S. & Zou, Z. Toxicity of *Chimonanthus nitens* flower extracts to the golden apple snail, *Pomacea canaliculata*. *Pestic. Biochem. Physiol.* **160**, 136–145 (2019). 31519248 10.1016/j.pestbp.2019.07.015

[61] Parolini, M., Ganzaroli, A. & Bacenetti, J. Earthworm as an alternative protein source in poultry and fish farming: Current applications and future perspectives. *Sci. Total Environ.* **734**, 139460 (2020). 32454339 10.1016/j.scitotenv.2020.139460

[62] Sadegh, H. et al. The role of nanomaterials as effective adsorbents and their applications in wastewater treatment. *J. Nanostruct. Chem.* **7**(1), 1–14 (2017).

[63] Arafa, N. H., Shehata, M. R. & Mohamed, A. S. Protective role of ovothiol-a against muscle and kidney injuries in obese rats. *Curr. Chem. Biol.* **18**(1), 30–45 (2024).

[64] Edwards, C. A. & Arancon, N. Q. *Earthworm Morphology* 1–31 (Springer, 2022).

[65] Courtois, P. et al. Accumulation, speciation and localization of silver nanoparticles in the earthworm *Eisenia fetida*. *Environ. Sci. Pollut. Res. Int.* **28**(4), 3756–3765 (2021). 32270459 10.1007/s11356-020-08548-z

[66] Azeez, O. I., Meintjes, R. & Chamunorwa, J. P. Fat body, fat pad and adipose tissues in invertebrates and vertebrates: The nexus. *Lipids Health Dis* **13**, 71 (2014). 24758278 10.1186/1476-511X-13-71 PMC4005007

[67] Arafa, N. H., Shehata, M. R. & Mohamed, A. S. Ovothiol-A mitigates high-fat diet-induced non-alcoholic fatty liver disease in rats. *Curr. Bioact. Compd.* **20**(8), 12–21 (2024).

[68] Zhang, L., Shi, Y., Liang, B. & Li, X. An overview of the cholesterol metabolism and its proinflammatory role in the development of MASLD. *Hepatol. Commun.* **8**(5), e0434 (2024). 38696365 10.1097/HC9.0000000000000434 PMC11068152

[69] Nandurkar, H. & Zambare, S. Comparative study of acute and chronic exposure of chloramphenicol on total lipid contents in different tissues of model animals, *Lamellidens corrianus* (Lea) and *Parreysia cylindrica* (Annandale and Prashad). *Int. Multidiscip. Res. J.* **2**(3), 33–35 (2012).

[70] Akazawa, S.-I. et al. High-pressure tolerance of earthworm fibrinolytic and digestive enzymes. *J. Biosci. Bioeng.* **125**(2), 155–159 (2018). 28916302 10.1016/j.jbiosc.2017.08.011

[71] Kiyasudeen, K., Ibrahim, M. H., Quaik, S. & Ismail, S. A. *Prospects of Organic Waste Management and the Significance of Earthworms* (Springer, 2015).

[72] de Souza, P. M. & de Oliveira Magalhães, P. Application of microbial α-amylase in industry—A review. *Braz. J. Microbiol.* **41**(4), 850–861 (2010). 24031565 10.1590/S1517-83822010000400004 PMC3769773

[73] Gugliucci, A. The chylomicron saga: Time to focus on postprandial metabolism. *Front. Endocrinol.* **14**, 1322869 (2023). 10.3389/fendo.2023.1322869 PMC10830840 38303975

[74] Singh, A. & Satheeshkumar, P. Reactive oxygen species (ROS) and ROS scavengers in plant abiotic stress response. In *Stress Biology in Photosynthetic Organisms: Molecular Insights and Cellular Responses* 41–63 (Springer, 2024).

[75] Hassan, M. A. T., Soliman, A. M. & Mohamed, A. S. The therapeutic potency of silver/chitosan, silver/saponin and chitosan/saponin nanocomposites on ethanol-induced gastric ulcers in wistar rats. *Recent Adv. Inflam. Allergy Drug Discov.* **18**(2), 115–128 (2024). 10.2174/0127722708283559240405075921 38629380

[76] Sies, H. & Jones, D. P. Reactive oxygen species (ROS) as pleiotropic physiological signalling agents. *Nat. Rev. Mol. Cell Biol.* **21**(7), 363–383 (2020). 32231263 10.1038/s41580-020-0230-3

[77] Jomova, K. et al. Reactive oxygen species, toxicity, oxidative stress, and antioxidants: Chronic diseases and aging. *Arch. Toxicol.* **97**(10), 2499–2574 (2023). 37597078 10.1007/s00204-023-03562-9 PMC10475008

[78] Sayed, F. A. Z., Mohamed, A. S. & Fahmy, H. M. Anti-cancer and safety profiles of doxorubicin-loaded kaolinite against ehrlich solid tumor. *Egypt. J. Chem.* **68**(1), 507–518 (2025).

[79] Khalaf, M. L., Soliman, A. M., Fahmy, S. R. & Mohamed, A. S. Anti-thrombotic mechanisms of echinochrome a on arterial thrombosis in rats: In-silico, in-vitro and in-vivo studies. *Cardiovasc. Hematol. Agents Med. Chem.* **23**(12), 143–160 (2025). 10.2174/0118715257332064241104114546 39533475

[80] Salehi, B. et al. Antioxidants: Positive or negative actors?. *Biomolecules* **8**(4), 124 (2018). 30366441 10.3390/biom8040124 PMC6316255

[81] Abd El Aziz, Y. E., Soliman, A. M., Fahmy, S. R. & Mohamed, A. S. Clove aqueous extract alleviates acute kidney injury induced by folic acid in rats. *Curr. Chem. Biol.* **18**(2), 104–112 (2024).

[82] Panova, I. G. & Tatikolov, A. S. Endogenous and exogenous antioxidants as agents preventing the negative effects of contrast media (contrast-induced nephropathy). *Pharmaceuticals (Basel)* **16**(8), 1077 (2023). 37630992 10.3390/ph16081077 PMC10458090

[83] Mohamed, A. S. Echinochrome exhibits antitumor activity against ehrlich ascites carcinoma in Swiss albino mice. *Nutr. Cancer* **73**(1), 124–132 (2021). 32151164 10.1080/01635581.2020.1737152

[84] Mohamed, A. T. A. E., Ragheb, M. A., Shehata, M. R. & Mohamed, A. S. In vivo cardioprotective effect of zinc oxide nanoparticles against doxorubicin-induced myocardial infarction by enhancing the antioxidant system and nitric oxide production. *J. Trace Elem. Med Biol.* **86**, 127516 (2024). 39226872 10.1016/j.jtemb.2024.127516

[85] Fath-All, A. A. et al. Efficacy of yeast-mediated SeNPs on gastric ulcer healing and gut microbiota dysbiosis in male albino rats. *Tissue Cell* **96**, 102953 (2025). 40334393 10.1016/j.tice.2025.102953

[86] Kotb, M. A. et al. Chrysin-loaded PLGA nanoparticles alleviate the implantation of endometriotic lesions via attenuation of peritoneal inflammation and downregulating NF-κB activation-driven expression of angiogenic factors. *J. Drug Deliv. Sci. Technol.* **109**, 106984 (2025).

[87] Moustafa, M. A., Mohamed, A. S., Dakrory, A. I. & Abdelaziz, M. H. Lepidium sativum extract alleviates reproductive and developmental toxicity in polycystic ovary syndrome induced by letrozole and high-fat diet in rats. *Reprod. Sci. (Thousand Oaks, Calif.)* **32**(4), 1338–1361 (2025). 10.1007/s43032-025-01820-y PMC11978546 40048056

[88] Fahmy, S. R., Abdel-Ghaffar, F., Bakry, F. A. & Sayed, D. A. Ecotoxicological effect of sublethal exposure to zinc oxide nanoparticles on freshwater snail *Biomphalaria alexandrina*. *Arch. Environ. Contam. Toxicol.* **67**(2), 192–202 (2014). 24736985 10.1007/s00244-014-0020-z

[89] Manna, S., Ray, A., Mukherjee, S., Ray, M. & Ray, S. Nano zinc oxide induced lipid peroxidation, oxidative stress, genotoxicity, phagocytic alteration, and detoxification response in the coelomocytes of an anecic earthworm of India. *Appl. Soil. Ecol.* **190**, 105024 (2023).

[90] García-Arcos, J. M., Jha, A., Waterman, C. M. & Piel, M. Blebology: Principles of bleb-based migration. *Trends Cell Biol.* **34**(10), 838–853 (2024). 38538441 10.1016/j.tcb.2024.02.009 PMC11424778

[91] Pacheco, I. & Buzea, C. Nanomaterials and nanocomposites: Classification and toxicity. In *Handbook of Nanomaterials Nanocomposites for Energy Environmental Applications*, 1–38 (2020).

